# Investigation of the Relationship Between University Students' Nomophobia and Psychological Distress: A Cross-sectional Study

**DOI:** 10.7150/ijms.112738

**Published:** 2025-06-23

**Authors:** Rakesh Kumar, Aisha Osman Yousif, Mohammed Ismail Humaida

**Affiliations:** 1 Department of Health Management, College of Public Health and Health Informatics, University of Ha'il, Ha'il, Saudi Arabia.; 2 Department of Public Health, College of Public Health and Health Informatics, University of Ha'il, Ha'il, Saudi Arabia.

**Keywords:** nomophobia, depression, anxiety, stress, mobile usage, Saudi Arabia

## Abstract

**Background:** This study investigated the relationship between nomophobia, psychological distress, and demographic factors among students.

**Methods:** This cross-sectional study used a convenience sampling approach to collect data from 723 respondents. The study participants were university students from the University of Ha'il. Distress factors were measured as dependent variables using the Depression Anxiety Stress Scale (DASS)-21, while nomophobia was the independent variable measured using the Nomophobia Questionnaire (NMP-Q). The relationships were assessed using Pearson's correlation, whereas the relationship power of these factors was assessed using hierarchical regression.

**Results:** The study results revealed that four dimensions of nomophobia were significantly correlated with depression, anxiety, stress, and overall DASS-21 scores (p < 0.01). Additionally, nomophobia was significantly associated with distress factors (depression: β = 0.11, p-value < 0.01, anxiety: β = 0.11, p-value < 0.01, stress: β = 0.08, p-value < 0.01). Mobile usage was also significantly associated with the same distress factors (depression: β = 1.67, p-value < 0.01, anxiety: β = 1.65, p-value < 0.01, stress: β = 1.65, p-value < 0.01). Additionally, gender was associated with anxiety (β = 2.75, p-value < 0.01,).

**Conclusion:** Nomophobia significantly exacerbates distress and is a leading cause of stress, anxiety, and depression. The study found that high mobile phone use significantly contributes to psychological distress, which leads to low academic performance, which—in turn—further increases distress.

## Introduction

The widespread use of smartphones has led to nomophobia—the fear of being without a smartphone. This phenomenon has gained attention owing to increasing smartphone dependency. University students are particularly affected, as they rely on smartphones for academic activities, including accessing study materials, conducting research, and collaborating with peers. Additionally, smartphones play a crucial role in social networking, enabling students to engage with friends, stay updated, and participate in online communities. However, excessive smartphone use has been linked to mental health concerns, raising alarm about its association with psychological well-being. Studies have shown that smartphone addiction is linked to increased levels of distress factors 1. Similarly, constant notifications and reliance on social validation contribute to psychological distress 2. Moreover, excessive smartphone use disrupts sleep patterns, reduces productivity, and encourages social withdrawal 3,4. These factors raise mental health concerns among university students 5. As smartphone addiction increases, researchers and mental health experts have become concerned about its long-term impact, particularly on students, given their stage of development.

In Saudi Arabia—particularly in the Hail region—smartphone usage has rapidly increased 6, becoming an essential part of daily life for university students. This increase—coupled with academic pressures and societal pressures—can contribute to greater vulnerability to nomophobia and related psychological distress. Yet little research has explored the problem in the University of Ha'il population. An understanding of the relationship between nomophobia and distress in this particular group will be key to overcoming possible mental health issues and informing regionally applicable interventions.

One of the most widely used tools for assessing psychological distress is the Depression Anxiety Stress Scale (DASS)-21. This tool measures and evaluates depression, anxiety, and stress levels 7. The scale comprises 21 items, with each subscale containing seven statements related to emotional distress. The DASS-21 is effective for detecting psychological distress in both clinical and nonclinical settings 8. Previous studies have examined factors such as age, gender, and academic performance in relation to smartphone addiction 9,10. Research suggests that university students experience greater smartphone-related distress due to their frequent engagement in digital activities 11.

The contribution of nomophobia to psychological distress depends largely on academic level and gender. Academic distress depends on the level of education such that students who have to face more advanced courses suffer most compared to others. Furthermore, there exist gender differences concerning the contribution of smartphone usage to psychological wellbeing. Women may endure emotional distress due to smartphone addiction, whereas men may endure social isolation, and thereby distress. Studies show that gender also plays a role in how smartphone use affects psychological distress 12,13. Women often experience more anxiety and stress due to social media engagement and social comparison with others of their gender 14-16. In contrast, men may be more prone to gaming addiction 17,18. While several studies have explored the relationship between nomophobia and psychological distress—including its depression, anxiety, and stress 19,20, many have focused on individual distress factors. This study expands the previous research by using the entire DASS-21 scale to capture the impact of nomophobia on all the dimensions of distress. Taking all of them together provides a clear idea of the role of nomophobia in the wellbeing of university students.

Previous studies have explored the link between nomophobia and psychological distress using the DASS-21 scale 21, 22. But they have not typically controlled for the academic level or gender, nor focused on the specific context of the University of Ha'il. This research fills these gaps by controlling them and focusing on the region of Hail. Incorporating academic level, gender, and regional context into the literature sheds new light on the relationship between nomophobia and psychological distress. Hence, this research will investigate the relationship between nomophobia and depression, anxiety, and stress in University of Ha'il students and contribute to strategies that mitigate smartphone-induced psychological distress.

## Material and Methods

### Study Design and Participants

This cross-sectional study used a convenience sampling approach for collecting survey data from University of Ha'il students' between 21/01/2025 and 10/02/2025. The inclusion criteria were enrolment as an undergraduate or postgraduate student, daily smartphone use, and agreement to participate in the study. Students with a history of mental health disorders, undergoing psychiatric treatment, or using prescribed psychological medications were excluded. Informed consent was obtained before collecting data from the participants. The participants' anonymity and privacy were preserved.

### Sampling

The research site for the University of Ha'il was chosen due to the prevalence of smartphones in the area and the specific scholarly and cultural pressures the Saudi students have to endure. The Hail region, with rapidly increasing smartphone usage, experienced high digital activity among the university students potentially vulnerable to nomophobia and psychological distress. Beyond the prevalence of smartphone usage, scholarly and cultural practices in the region make it hard for the students. Such challenges make the University of Ha'il a relevant site to examine the interrelationship between nomophobia and distress. Gender and scholarly status were also factors in the research since they were known to affect the expression of smartphone addiction and associated distress among students.

### Survey Instrument

The study used Nomophobia Questionnaire (NMP-Q) developed by Yildirim and Correia 23 to assess nomophobia. It comprises four dimensions: not being able to communicate, losing connectedness, not being able to access information, and giving up convenience. A seven-point scale ranging from strongly disagree to strongly agree was used to rate the 20-item responses for the NMP-Q. Higher scores indicated greater levels of nomophobia. Distress factors were measured using the 21-item scale measuring depression, anxiety, and stress (DASS-21) developed by Lovibond and Lovibond 8. It comprises 21 items measured using a four-point scale ranging from 0 = never to 3 = almost always. Higher scores indicated greater distress levels. The Cronbach's alpha of the NMP-Q and DASS-21 was 0.946 and 0.951 respectively. We adopted survey items using standard scales from the English version. Each item/statement was also translated into the National Language (Arabic) to ensure clarity and accessibility for all the participants.

The demographic variables included in this study were age, gender, academic level, duration of mobile phone usage (hours per day), and living situation. These variables were included to assess their associations with nomophobia and psychological distress. Data were collected through an online survey shared through university platforms. Participants received a detailed description of the study before completing the questionnaire. The responses were anonymized and participation was voluntary. The survey took approximately 10-15 min to complete. Participants could withdraw at any stage without justification. The pre-tested translated questionnaire was administered to a pilot group of 12 individuals representing the target population before actual data collection. Pre-testing was conducted to ensure that the survey items were easy to read and understand and addressed the constructs intended. A few minor adjustments for clarity were made based on the participants' responses.

### Statistical Analysis

Data were analyzed using SPSS version 27 software. A demographic summary was generated using the frequency of participants' demographic details. Furthermore, descriptive statistics of the measures summarizing their means, standard deviations, and minimum and maximum values were also estimated. Additionally, Pearson's correlation analysis was used to examine the relationship between nomophobia and psychological distress factors (depression, anxiety, and stress). Finally, a hierarchical regression analysis was conducted to assess the associative role of nomophobia while controlling for demographic variables. P-values < 0.01, < 0.05, and < 0.10 were considered statistically highly significant, significant, and weakly significant, respectively.

## Results

### Demographic Analysis

In total, 723 students were surveyed. Table 1 presents the demographic characteristics of the respondents. The respondents were predominantly male, followed by female respondents. Most students were enrolled in the bachelor's program, with a smaller proportion in the master's program. In terms of age distribution, the majority of the participants were between 18-21 years, followed by 22--25 years, 26-30 years, and 31 years or older. Regarding mobile usage, most students reported using their phones for 5-6 hours or 7-8 hours daily. The majority of participants lived off-campus with their families, while smaller proportions lived alone, with roommates, or in an on-campus hostel.

### Descriptive Statistics for Measurements

Table 2 presents the descriptive statistics of nomophobia (NMP-Q) and distress factors (DASS-21). The results indicate that the participants exhibited moderately high levels of nomophobia, considering the possible range of scores. Among the dimensions, losing connectedness had the lowest mean score (M = 17.49, SD = 8.00), whereas giving up convenience had a relatively higher mean score (M = 22.47, SD = 7.84). The dimension of not being able to communicate showed a notably high mean score (M = 27.41, SD = 8.86), suggesting that the participants experienced a strong fear of being unable to communicate via smartphones.

Regarding distress factors, the total DASS-21 score (M = 41.10, SD = 30.50) suggested a moderate level of psychological distress. Among the three components, stress (M = 14.09, SD = 11.08) was slightly higher than depression (M = 13.53, SD = 10.27) and anxiety (M = 13.47, SD = 12.17). These findings suggest that students experienced moderate distress, with stress levels being the most prominent.

### Variable Relationship Analysis

Table 3 presents the Pearson correlation coefficients examining the relationship between nomophobia (NMP-Q) and distress factors (DASS-21) along with key demographic and behavioral variables. The findings indicate significant positive associations between nomophobia dimensions and distress factors. Specifically, all four dimensions of nomophobia—not being able to communicate, losing connectedness, not being able to access information, and giving up convenience—were significantly correlated with depression, anxiety, stress, and overall DASS-21 scores (*p* < 0.01). The strongest associations were observed between giving up convenience and total DASS-21 (*r* = .308, *p* < 0.01) and between NMP-Q total and total DASS-21 (*r* = .308, *p* < 0.01) scores, suggesting that higher nomophobia levels are linked with greater psychological distress.

Among the demographic variables, gender was significantly associated with anxiety (*r* = .127, *p* < 0.01) and overall DASS-21 scores (*r* = .092, *p* < 0.05), indicating that female participants reported higher anxiety levels. Mobile use showed a significant positive correlation with all the distress factors, highlighting its role in psychological distress. However, age, academic level, and living situation indicated no significant association with distress factors. Our results indicate that gender and academic level are significant factors in the relationship between nomophobia and psychological distress. Specifically, women reported higher levels of anxiety, which aligns with previous studies on gender differences in smartphone addiction and mental health 24.

Significant correlations were found between nomophobia and distress factors, with giving up convenience and not being able to access information showing the strongest associations. Higher mobile phone use was correlated with greater depression, anxiety, and stress. Women reported higher anxiety levels, whereas age, academic level, and living situation showed no significant relationships with distress factors. These findings suggest that reliance on smartphones, particularly for comfort and information, contributes to psychological distress.

### Associations of Depression, Anxiety, and Stress

The scores for depression are presented in Table 4. Depression levels were higher among individuals with greater mobile phone usage (*coefficient* = 2.43, *p* < 0.01) and among those experiencing higher levels of nomophobia (*coefficient* = 0.11, *p* < 0.01). Academic level indicated a negative correlation with depression (*coefficient* = -1.81, *p* < 0.10). However, age, gender, and living situation were not significantly associated with depression. The final model explained 12.2% of the variance in depression levels.

The scores for anxiety are presented in Table 5. Anxiety was significantly higher in women (*β* = 2.79, *p* < 0.01) and among individuals with greater mobile phone use (*β* = 2.43, *p* < 0.01) and nomophobia (*β* = 0.11, *p* < 0.01). Academic level was negatively associated with anxiety (*β* = -2.63, *p* < 0.05). However, age and living situation were not significantly associated with anxiety. The final model accounted for 10.6% of the variance in anxiety levels.

The scores for stress are presented in Table 6. Nomophobia (NMP-Q) and mobile phone use strongly enhanced stress levels, with a coefficient value of 0.09, and p-value of 0.01, and coefficient value of 2.28, and p-value of 0.01, respectively. Academic level had a negative association with stress (*β* = -2.60, *p* < 0.10), whereas age, gender, and living situation were not significantly associated. The final model explained 8.1% of the variance in the stress levels.

These findings reveal that nomophobia and mobile phone use are consistently associated with depression, anxiety, and stress—the three distress factors—whereas academic level adversely affects all three distress factors. Gender affects only anxiety levels in that women experience more symptoms of anxiety.

## Discussion

In this cross-sectional study, we found that nomophobia was significantly associated with depression, anxiety, and stress, along with other demographic factors such as academic level and phone usage. Importantly, nomophobia was found to have a strong correlation with psychological distress, in accordance with other studies that found a negative association with emotional well-being 1,24. Excessive use of cellular phones has been found to cause more psychological distress in university students; specifically, extensive phone usage and digital reliance have been found to induce emotional instability along with nervousness 25.

Our findings further identified that gender was significantly associated with anxiety as women reported higher anxiety than men as in other research that has established that women in general have greater vulnerability to anxiety disorders due to biological as well as psychosocial variables. It aligned with other research that has established that women in general have greater vulnerability to anxiety disorders due to biological as well as psychosocial variables 26.

Moreover, academic level was shown to have a negative impact on distress in that higher academic qualification was associated with decreased depression, anxiety, and stress levels. Our conjecture is that this may be caused by higher coping mechanisms and higher individual resilience levels with increased academic attainment 27. Mobile phone use was shown to be significantly associated with all three distress factors, in that higher levels of screen time have implications for causing mental illness by reducing face-to-face human interactions while increasing reliance on digital spaces 28.

The role of living conditions was not found to be significant in the association of distress, contrary to other research that has established that the environment plays a crucial role in emotional well-being 29. Alternatively, it might be that the coping mechanisms of individuals—rather than the living conditions themselves—play a more direct role in distress levels.

Our study highlights the significance of nomophobia in relation to psychological distress, establishing that measures to counter its link must be implemented. Mindfulness treatment and digital detox regimes have been shown to significantly mitigate psychological associations with smartphone overindulgence 30. Programs for awareness and early detection of susceptible individuals such as college students have the potential to neutralize the negative associations with nomophobia 31. Future research should explore how nomophobia contributes to depression, anxiety, and stress in other categories of participants and regions, helping to develop interventions that mitigate negative psychological associations.

Despite these results, our study has several limitations. First, due to the cross-sectional research design, we cannot determine the causality between distress variables and nomophobia. Long-term follow-up studies are required to confirm these associations over time. Second, our use of self-reported measures may introduce a response bias, in that individuals might have underreported or overreported their level of distress. Follow-up research should include physiological factors (like variations in heart rate) to provide quantitative indices of psychological distress. Third, in not having accounted for in our analysis for socioeconomic status or personality traits in their research—although these have been shown to have large associations with psychological distress—future research must explore these other variables to provide a more comprehensive explanation of distress determinants.

### Practical Implications and Future Research

Our findings call for digital wellness programs to reach individuals to create awareness of the psychological implications of intensive phone use 32. Mental awareness drives need to be integrated by organizations for their learners, particularly for students with intensive phone-use behaviors. Counseling that focuses on coping mechanisms for nomophobia must also be introduced—for instance—by advocating for off-line activities as well as face-to-face human interaction.

Future research should explore longitudinal links between nomophobia and mental well-being to establish causality. Cross-cultural research would further provide insight into how societal and cultural factors are associated with the relationship between mobile phone addiction and the level of distress. Research on coping and resilience in minimizing associations with nomophobia will further inform future interventions.

In conclusion, we determined that nomophobia is strongly associated with depression, anxiety, and stress, further underscoring the need for specific interventions to counter its association with mental well-being. Through prevention measures and digital literacy, individual's mobile phone use can be managed and digital-age psychological resilience can be cultivated.

## Conclusion

Nomophobia significantly affects distress in terms of stress, anxiety, and depression. This study identified three key contributing factors to distress levels among university students: (1) high mobile phone use was a contributing factor to psychological distress; (2) low academic levels were associated with higher distress; and (3) nomophobia was a leading factor for stress, anxiety, and depression.

The outcomes also demonstrate the direct relationship of gender with anxiety levels, with females showing higher anxiety symptoms than males. The outcomes verify the research goals, as the prevalence of nomophobia and the use of mobile phones consistently relates to psychological distress, while gender and academic level account for the variance in outcomes of distress.

This study sheds considerable light on the relationship between nomophobia, psychological distress, and the sociodemographic factors of gender and academic level. By considering the complete DASS-21 scale and a broad spectrum of distress factors, it better captures the impacts of smartphone addiction on the psychological health of university students. This study extends previous studies insofar as it considers gender and academic level and maintains a comprehensive approach in the understanding of nomophobia and psychological distress.

This research indicates the necessity to create targeted mental health strategies and raise awareness for healthy mobile usage in order to reduce distress in students. Mitigating and coping with nomophobia and its psychological connections will be significant in the development of student well-being.

Future research should explore additional factors contributing to psychological distress and investigate effective interventions to minimize the adverse associations between nomophobia and mental health. Furthermore, larger-scale studies across diverse regions are recommended to examine potential regional trends, thereby enhancing the generalizability and broader applicability of the findings.

## Figures and Tables

**Table 1:** Demographic Characteristics of the Participants

Characteristics	Categories	N	%
Gender	Male	452	62.5%
	Female	271	37.5%
Education	Bachler's Program	625	86.44%
	Master's Program	98	13.6%
Age (years)	18-21	473	65.4%
	22-25	116	16.0%
	26-30	62	8.6%
	31 and above	72	10.0%
Mobile Phone Use (in hours)	1-2	19	2.6%
	3-4	171	23.7%
	5-6	271	37.5%
	7-8	262	36.2%
Living Situation	On-campus Hostel	10	1.4%
	Off-campus with family	586	81.1%
	Off-campus with roommates	52	7.2%
	Alone	75	10.4%

**Table 2:** Descriptive Statistics of the Variables

Variables	Min	Max	Mean	SD
**Nomophobia (NMP-Q)**				
Not being able to Communicate	6.00	42.00	27.41	8.86
Losing Connectedness	5.00	35.00	17.49	8.00
Not being able to Access Information	4.00	28.00	18.39	6.31
Giving up Convenience	5.00	35.00	22.47	7.84
Total Score	20.00	140.00	85.76	26.67
**Distress Factors (DASS-21)**				
Depression	0.00	42.00	13.53	10.27
Anxiety	0.00	42.00	13.47	12.17
Stress	0.00	42.00	14.09	11.08
Total Score	0.00	126.00	41.10	30.50

**Note: SD =** Standard Deviation

**Table 3:** The Variable Correlations (NMP-Q and DASS-21)

Variables	Depression	Anxiety	Stress	DASS-21 Total
Not being able to Communicate	.219***	.205***	.164***	.215***
Losing Connectedness	.300***	.223***	.183***	.256***
Not being able to Access Information	.288***	.271***	.235***	.291***
Giving up Convenience	292***	.277***	.273***	.308***
NMP-Q Total	.317***	.281***	.245***	.308***
Age	0.035	0.018	0.013	0.024
Gender	0.059	.127***	0.059	.092**
Academic Level	0.007	-0.013	-0.022	-0.011
Mobile Phone Use	.200***	.171***	.172***	.198***
Living Situation	0.010	-0.04	0.004	-0.012

Note: NMP-Q = Nomophobia Questionnaire, DASS-21 = the 21 items of the Depression Anxiety and Stress Scale * p<0.10, ** p<0.05, *** p<0.01

**Table 4:** Depression Scores Measured using Hierarchical Regression

Variables	Step-1	Step-2	Step-3	Step-4	Step-5	Step-6	Tol	VIF
Age	0.362	0.350	1.38*	1.39*	1.38*	0.822	1.00	1.00
Gender		1.233	1.31*	1.121	1.170	0.924	1.00	1.00
Academic Level			-1.635	-1.81*	-1.810*	-1.125	0.99	1.01
Mobile Phone Use				2.43***	2.43***	1.67***	0.95	1.05
Living Situation					0.266	0.539	0.95	1.05
NMP-Q						0.11***	0.88	1.14
Constant	12.94***	11.26***	11.84***	4.87***	4.21*	-3.16***		
R	0.035	0.068	0.087	0.216	0.217	0.35		
R-Square	0.001	0.005	0.008	0.047	0.047	0.122		
Adj-R-Square	0.000	0.002	0.003	0.041	0.040	0.115		
R-Square Change	0.001	0.003	0.003	0.039	0.000	0.075		
F	0.902	1.676	1.842	8.78***	7.06***	16.62***		
F-Change	0.902	2.448	2.169	29.374	0.212	61.427		
Sig-F	0.343	0.188	0.138	0.000	0.000	0.000		
Sig-F Change	0.343	0.118	0.141	0.000	0.000	0.000		

Note: NMP-Q = Nomophobia Questionnaire, DASS-21 = the 21 items of the Depression Anxiety and Stress Scale * p<0.10, ** p<0.05, *** p<0.01

**Table 5:** Anxiety Scores Measured using Hierarchical Regression

Variables	Step-1	Step-2	Step-3	Step-4	Step-5	Step-6	Tol	VIF
Age	0.220	0.189	1.73*	1.74*	1.77*	1.203	1.00	1.00
Gender		3.19***	3.31***	3.12***	3.04***	2.79***	0.98	1.02
Academic Level			-2.440*	-2.623**	-2.637**	-1.941	0.98	1.02
Mobile Phone Use				2.44***	2.43***	1.65***	0.95	1.05
Living Situation					-0.446	-0.166	0.95	1.05
NMP-Q						0.11***	0.89	1.12
Constant	13.11***	8.77***	9.64***	2.648	3.757	-3.783		
R	0.018	0.128	0.146	0.222	0.223	0.325		
R-Square	0.000	0.016	0.021	0.049	0.050	0.106		
Adj-R-Square	-0.001	0.014	0.017	0.044	0.043	0.098		
R-Square Change	0.000	0.016	0.005	0.028	0.001	0.056		
F	0.237	6.03***	5.20***	9.27***	7.50***	14.13***		
F-Change	0.237	11.818	3.496	21.060	0.426	44.987		
Sig-F	0.626	0.003	0.001	0.001	0.001	0.001		
Sig-F Change	0.626	0.001	0.062	0.000	0.514	0.000		

Note: NMP-Q = Nomophobia Questionnaire, DASS-21 = the 21 items of the Depression Anxiety and Stress Scale * p<0.10, ** p<0.05, *** p<0.01

**Table 6:** Stress Scores Measured using Hierarchical Regression

Variables	Step-1	Step-2	Step-3	Step-4	Step-5	Step-6	Tol	VIF
Age	0.147	0.134	1.670*	1.682*	1.669**	1.221	1.00	1.00
Gender		1.336	1.452*	1.274	1.310	1.111	1.00	1.00
Academic Level			-2.434**	-2.601*	-2.595**	-2.046*	0.99	1.01
Mobile Phone Use				2.277***	2.279***	1.658***	0.96	1.04
Living Situation					0.196	0.417	0.96	1.04
NMP-Q						0.088***	0.92	1.09
Constant	13.85***	12.03***	12.90***	6.367**	5.880**	-0.068		
R	0.013	0.06	0.096	0.197	0.197	0.285		
R-Square	0.000	0.004	0.009	0.039	0.039	0.081		
Adj-R-Square	-0.001	0.001	0.005	0.033	0.032	0.073		
R-Square Change	0.000	0.003	0.006	0.029	0.000	0.042		
F	0.127	1.296	2.25*	7.22***	5.79***	10.53***		
F-Change	0.127	2.47	4.14	21.95	0.09	32.91		
Sig-F	0.722	0.274	0.082	0.001	0.001	0.001		
Sig-F Change	0.722	0.117	0.042	0.000	0.000	0.000		

Note: NMP-Q = Nomophobia Questionnaire, DASS-21 = the 21 items of the Depression Anxiety and Stress Scale * p<0.10, ** p<0.05, *** p<0.01
